# Supplementary material for: HIVprotI: an integrated web based platform for prediction and design of HIV proteins inhibitors
Source: J Cheminform. 2018 Mar 9;10:12. doi: 10.1186/s13321-018-0266-y (PMC5845081; doi:10.1186/s13321-018-0266-y)
Supplement: Supplementary file 2 — Additional file 2. Source code of HIVProtI web server. [file 13321_2018_266_MOESM2_ESM.zip › HIVprotI_Source-code/hivprotI/weka-3-6-6/doc/weka/gui/beans/ClassValuePicker.html]

ClassValuePicker


---


|  |  |  |
| --- | --- | --- |
| **Overview** \| **Package** \| **Class** \| **Tree** \| **Deprecated** \| **Index** \| **Help** \| **Weka's home** | |  |
| **PREV CLASS**   **NEXT CLASS** | **FRAMES**    **NO FRAMES**     **All Classes** |  |
| SUMMARY: NESTED \| FIELD \| CONSTR \| METHOD | DETAIL: FIELD \| CONSTR \| METHOD |  |


---


## weka.gui.beans Class ClassValuePicker

```
java.lang.Object
  java.awt.Component
      java.awt.Container
          javax.swing.JComponent
              javax.swing.JPanel
                  weka.gui.beans.ClassValuePicker
```

**All Implemented Interfaces:**: java.awt.image.ImageObserver, java.awt.MenuContainer, java.io.Serializable, java.util.EventListener, javax.accessibility.Accessible, BeanCommon, DataSourceListener, EventConstraints, StructureProducer, Visible

---

``` public class ClassValuePicker extends javax.swing.JPanel implements Visible, DataSourceListener, BeanCommon, EventConstraints, java.io.Serializable, StructureProducer ```

**Version:**
:   $Revision: 7439 $

**Author:**
:   Mark Hall

**See Also:**: Serialized Form

---

**Nested Class Summary**

**Nested classes/interfaces inherited from class javax.swing.JComponent**

`javax.swing.JComponent.AccessibleJComponent`

**Nested classes/interfaces inherited from class java.awt.Component**

`java.awt.Component.BaselineResizeBehavior`


**Field Summary**

**Fields inherited from class javax.swing.JComponent**

`TOOL_TIP_TEXT_KEY, UNDEFINED_CONDITION, WHEN_ANCESTOR_OF_FOCUSED_COMPONENT, WHEN_FOCUSED, WHEN_IN_FOCUSED_WINDOW`

**Fields inherited from class java.awt.Component**

`BOTTOM_ALIGNMENT, CENTER_ALIGNMENT, LEFT_ALIGNMENT, RIGHT_ALIGNMENT, TOP_ALIGNMENT`

**Fields inherited from interface java.awt.image.ImageObserver**

`ABORT, ALLBITS, ERROR, FRAMEBITS, HEIGHT, PROPERTIES, SOMEBITS, WIDTH`


**Constructor Summary**

`ClassValuePicker()`


| **Method Summary** | |
| --- | --- |
| `void` | `acceptDataSet(DataSetEvent e)` |
| `void` | `addDataFormatListener(DataFormatListener dfl)` |
| `void` | `addDataSourceListener(DataSourceListener tsl)` |
| `boolean` | `connectionAllowed(java.beans.EventSetDescriptor esd)`             Returns true if, at this time, the object will accept a connection according to the supplied EventSetDescriptor |
| `boolean` | `connectionAllowed(java.lang.String eventName)`             Returns true if, at this time, the object will accept a connection according to the supplied event name |
| `void` | `connectionNotification(java.lang.String eventName, java.lang.Object source)`             Notify this object that it has been registered as a listener with a source with respect to the supplied event name |
| `void` | `disconnectionNotification(java.lang.String eventName, java.lang.Object source)`             Notify this object that it has been deregistered as a listener with a source with respect to the supplied event name |
| `boolean` | `eventGeneratable(java.lang.String eventName)`             Returns true, if at the current time, the named event could be generated. |
| `java.lang.String` | `getClassValue()`             Gets the class value considered to be the "positive" class value. |
| `Instances` | `getConnectedFormat()`             Returns the structure of the incoming instances (if any) |
| `java.lang.String` | `getCustomName()`             Get the custom (descriptive) name for this bean (if one has been set) |
| `Instances` | `getStructure(java.lang.String eventName)`             Get the structure of the output encapsulated in the named event. |
| `BeanVisual` | `getVisual()`             Get the visual representation |
| `java.lang.String` | `globalInfo()`             Global info for this bean |
| `boolean` | `isBusy()`             Returns true if. |
| `void` | `removeDataFormatListener(DataFormatListener dfl)` |
| `void` | `removeDataSourceListener(DataSourceListener tsl)` |
| `void` | `setClassValue(java.lang.String value)`             Set the class value index considered to be the "positive" class value. |
| `void` | `setCustomName(java.lang.String name)`             Set a custom (descriptive) name for this bean |
| `void` | `setLog(Logger logger)`             Set a logger |
| `void` | `setVisual(BeanVisual newVisual)`             Set a new visual representation |
| `void` | `stop()`             Stop any processing that the bean might be doing. |
| `void` | `useDefaultVisual()`             Use the default visual representation |

**Methods inherited from class javax.swing.JPanel**

`getAccessibleContext, getUI, getUIClassID, setUI, updateUI`

**Methods inherited from class javax.swing.JComponent**

`addAncestorListener, addNotify, addVetoableChangeListener, computeVisibleRect, contains, createToolTip, disable, enable, firePropertyChange, firePropertyChange, firePropertyChange, getActionForKeyStroke, getActionMap, getAlignmentX, getAlignmentY, getAncestorListeners, getAutoscrolls, getBaseline, getBaselineResizeBehavior, getBorder, getBounds, getClientProperty, getComponentPopupMenu, getConditionForKeyStroke, getDebugGraphicsOptions, getDefaultLocale, getFontMetrics, getGraphics, getHeight, getInheritsPopupMenu, getInputMap, getInputMap, getInputVerifier, getInsets, getInsets, getListeners, getLocation, getMaximumSize, getMinimumSize, getNextFocusableComponent, getPopupLocation, getPreferredSize, getRegisteredKeyStrokes, getRootPane, getSize, getToolTipLocation, getToolTipText, getToolTipText, getTopLevelAncestor, getTransferHandler, getVerifyInputWhenFocusTarget, getVetoableChangeListeners, getVisibleRect, getWidth, getX, getY, grabFocus, isDoubleBuffered, isLightweightComponent, isManagingFocus, isOpaque, isOptimizedDrawingEnabled, isPaintingForPrint, isPaintingTile, isRequestFocusEnabled, isValidateRoot, paint, paintImmediately, paintImmediately, print, printAll, putClientProperty, registerKeyboardAction, registerKeyboardAction, removeAncestorListener, removeNotify, removeVetoableChangeListener, repaint, repaint, requestDefaultFocus, requestFocus, requestFocus, requestFocusInWindow, resetKeyboardActions, reshape, revalidate, scrollRectToVisible, setActionMap, setAlignmentX, setAlignmentY, setAutoscrolls, setBackground, setBorder, setComponentPopupMenu, setDebugGraphicsOptions, setDefaultLocale, setDoubleBuffered, setEnabled, setFocusTraversalKeys, setFont, setForeground, setInheritsPopupMenu, setInputMap, setInputVerifier, setMaximumSize, setMinimumSize, setNextFocusableComponent, setOpaque, setPreferredSize, setRequestFocusEnabled, setToolTipText, setTransferHandler, setVerifyInputWhenFocusTarget, setVisible, unregisterKeyboardAction, update`

**Methods inherited from class java.awt.Container**

`add, add, add, add, add, addContainerListener, addPropertyChangeListener, addPropertyChangeListener, applyComponentOrientation, areFocusTraversalKeysSet, countComponents, deliverEvent, doLayout, findComponentAt, findComponentAt, getComponent, getComponentAt, getComponentAt, getComponentCount, getComponents, getComponentZOrder, getContainerListeners, getFocusTraversalKeys, getFocusTraversalPolicy, getLayout, getMousePosition, insets, invalidate, isAncestorOf, isFocusCycleRoot, isFocusCycleRoot, isFocusTraversalPolicyProvider, isFocusTraversalPolicySet, layout, list, list, locate, minimumSize, paintComponents, preferredSize, printComponents, remove, remove, removeAll, removeContainerListener, setComponentZOrder, setFocusCycleRoot, setFocusTraversalPolicy, setFocusTraversalPolicyProvider, setLayout, transferFocusBackward, transferFocusDownCycle, validate`

**Methods inherited from class java.awt.Component**

`action, add, addComponentListener, addFocusListener, addHierarchyBoundsListener, addHierarchyListener, addInputMethodListener, addKeyListener, addMouseListener, addMouseMotionListener, addMouseWheelListener, bounds, checkImage, checkImage, contains, createImage, createImage, createVolatileImage, createVolatileImage, dispatchEvent, enable, enableInputMethods, firePropertyChange, firePropertyChange, firePropertyChange, firePropertyChange, firePropertyChange, getBackground, getBounds, getColorModel, getComponentListeners, getComponentOrientation, getCursor, getDropTarget, getFocusCycleRootAncestor, getFocusListeners, getFocusTraversalKeysEnabled, getFont, getForeground, getGraphicsConfiguration, getHierarchyBoundsListeners, getHierarchyListeners, getIgnoreRepaint, getInputContext, getInputMethodListeners, getInputMethodRequests, getKeyListeners, getLocale, getLocation, getLocationOnScreen, getMouseListeners, getMouseMotionListeners, getMousePosition, getMouseWheelListeners, getName, getParent, getPeer, getPropertyChangeListeners, getPropertyChangeListeners, getSize, getToolkit, getTreeLock, gotFocus, handleEvent, hasFocus, hide, imageUpdate, inside, isBackgroundSet, isCursorSet, isDisplayable, isEnabled, isFocusable, isFocusOwner, isFocusTraversable, isFontSet, isForegroundSet, isLightweight, isMaximumSizeSet, isMinimumSizeSet, isPreferredSizeSet, isShowing, isValid, isVisible, keyDown, keyUp, list, list, list, location, lostFocus, mouseDown, mouseDrag, mouseEnter, mouseExit, mouseMove, mouseUp, move, nextFocus, paintAll, postEvent, prepareImage, prepareImage, remove, removeComponentListener, removeFocusListener, removeHierarchyBoundsListener, removeHierarchyListener, removeInputMethodListener, removeKeyListener, removeMouseListener, removeMouseMotionListener, removeMouseWheelListener, removePropertyChangeListener, removePropertyChangeListener, repaint, repaint, repaint, resize, resize, setBounds, setBounds, setComponentOrientation, setCursor, setDropTarget, setFocusable, setFocusTraversalKeysEnabled, setIgnoreRepaint, setLocale, setLocation, setLocation, setName, setSize, setSize, show, show, size, toString, transferFocus, transferFocusUpCycle`

**Methods inherited from class java.lang.Object**

`equals, getClass, hashCode, notify, notifyAll, wait, wait, wait`

**Constructor Detail**

### ClassValuePicker

```
public ClassValuePicker()
```


**Method Detail**

### globalInfo

```
public java.lang.String globalInfo()
```

:   Global info for this bean

    :   **Returns:**: a `String` value

---


### setCustomName

```
public void setCustomName(java.lang.String name)
```

:   Set a custom (descriptive) name for this bean

    :   **Specified by:**: `setCustomName` in interface `BeanCommon`
    :   **Parameters:**: `name` - the name to use

---


### getCustomName

```
public java.lang.String getCustomName()
```

:   Get the custom (descriptive) name for this bean (if one has been set)

    :   **Specified by:**: `getCustomName` in interface `BeanCommon`
    :   **Returns:**: the custom name (or the default name)

---


### getStructure

```
public Instances getStructure(java.lang.String eventName)
```

:   **Description copied from interface: `StructureProducer`**
:   Get the structure of the output encapsulated in the named
    event. If the structure can't be determined in advance of
    seeing input, or this StructureProducer does not generate
    the named event, null should be returned.

    :   **Specified by:**: `getStructure` in interface `StructureProducer`
    :   **Parameters:**: `eventName` - the name of the output event that encapsulates the requested output. **Returns:**: the structure of the output encapsulated in the named event or null if it can't be determined in advance of seeing input or the named event is not generated by this StructureProduce.

---


### getConnectedFormat

```
public Instances getConnectedFormat()
```

:   Returns the structure of the incoming instances (if any)

    :   **Returns:**: an `Instances` value

---


### setClassValue

```
public void setClassValue(java.lang.String value)
```

:   Set the class value index considered to be the "positive"
    class value.

    :   **Parameters:**: `index` - the class value index to use

---


### getClassValue

```
public java.lang.String getClassValue()
```

:   Gets the class value considered to be the "positive"
    class value.

    :   **Returns:**: the class value index

---


### acceptDataSet

```
public void acceptDataSet(DataSetEvent e)
```

:   **Specified by:**: `acceptDataSet` in interface `DataSourceListener`

---


### addDataSourceListener

```
public void addDataSourceListener(DataSourceListener tsl)
```

---


### removeDataSourceListener

```
public void removeDataSourceListener(DataSourceListener tsl)
```

---


### addDataFormatListener

```
public void addDataFormatListener(DataFormatListener dfl)
```

---


### removeDataFormatListener

```
public void removeDataFormatListener(DataFormatListener dfl)
```

---


### setVisual

```
public void setVisual(BeanVisual newVisual)
```

:   **Description copied from interface: `Visible`**
:   Set a new visual representation

    :   **Specified by:**: `setVisual` in interface `Visible`
    :   **Parameters:**: `newVisual` - a `BeanVisual` value

---


### getVisual

```
public BeanVisual getVisual()
```

:   **Description copied from interface: `Visible`**
:   Get the visual representation

    :   **Specified by:**: `getVisual` in interface `Visible`
    :   **Returns:**: a `BeanVisual` value

---


### useDefaultVisual

```
public void useDefaultVisual()
```

:   **Description copied from interface: `Visible`**
:   Use the default visual representation

    :   **Specified by:**: `useDefaultVisual` in interface `Visible`

---


### connectionAllowed

```
public boolean connectionAllowed(java.lang.String eventName)
```

:   Returns true if, at this time,
    the object will accept a connection according to the supplied
    event name

    :   **Specified by:**: `connectionAllowed` in interface `BeanCommon`
    :   **Parameters:**: `eventName` - the event **Returns:**: true if the object will accept a connection

---


### connectionAllowed

```
public boolean connectionAllowed(java.beans.EventSetDescriptor esd)
```

:   Returns true if, at this time,
    the object will accept a connection according to the supplied
    EventSetDescriptor

    :   **Specified by:**: `connectionAllowed` in interface `BeanCommon`
    :   **Parameters:**: `esd` - the EventSetDescriptor **Returns:**: true if the object will accept a connection

---


### connectionNotification

```
public void connectionNotification(java.lang.String eventName,
                                   java.lang.Object source)
```

:   Notify this object that it has been registered as a listener with
    a source with respect to the supplied event name

    :   **Specified by:**: `connectionNotification` in interface `BeanCommon`
    :   **Parameters:**: `eventName` - the event: `source` - the source with which this object has been registered as a listener

---


### disconnectionNotification

```
public void disconnectionNotification(java.lang.String eventName,
                                      java.lang.Object source)
```

:   Notify this object that it has been deregistered as a listener with
    a source with respect to the supplied event name

    :   **Specified by:**: `disconnectionNotification` in interface `BeanCommon`
    :   **Parameters:**: `eventName` - the event: `source` - the source with which this object has been registered as a listener

---


### setLog

```
public void setLog(Logger logger)
```

:   **Description copied from interface: `BeanCommon`**
:   Set a logger

    :   **Specified by:**: `setLog` in interface `BeanCommon`
    :   **Parameters:**: `logger` - a `weka.gui.Logger` value

---


### stop

```
public void stop()
```

:   **Description copied from interface: `BeanCommon`**
:   Stop any processing that the bean might be doing.

    :   **Specified by:**: `stop` in interface `BeanCommon`

---


### isBusy

```
public boolean isBusy()
```

:   Returns true if. at this time, the bean is busy with some
    (i.e. perhaps a worker thread is performing some calculation).

    :   **Specified by:**: `isBusy` in interface `BeanCommon`
    :   **Returns:**: true if the bean is busy.

---


### eventGeneratable

```
public boolean eventGeneratable(java.lang.String eventName)
```

:   Returns true, if at the current time, the named event could
    be generated. Assumes that the supplied event name is
    an event that could be generated by this bean

    :   **Specified by:**: `eventGeneratable` in interface `EventConstraints`
    :   **Parameters:**: `eventName` - the name of the event in question **Returns:**: true if the named event could be generated at this point in time


---


|  |  |  |
| --- | --- | --- |
| **Overview** \| **Package** \| **Class** \| **Tree** \| **Deprecated** \| **Index** \| **Help** \| **Weka's home** | |  |
| **PREV CLASS**   **NEXT CLASS** | **FRAMES**    **NO FRAMES**     **All Classes** |  |
| SUMMARY: NESTED \| FIELD \| CONSTR \| METHOD | DETAIL: FIELD \| CONSTR \| METHOD |  |


---
